# Swedish centenarian health – a nationwide, observational study on care utilization, drug use, morbidity, and mortality among Swedish centenarians in 1990 to 2022

**DOI:** 10.1186/s12877-025-06798-5

**Published:** 2025-11-25

**Authors:** Jimmy Lindberg, Yuge Zhang, Shunsuke Murata, Ingemar Kåreholt, Karin Modig

**Affiliations:** 1 Division of Social Work, Department of Culture and Society, Linköping University, Linköping, Sweden; 2 Unit of Epidemiology, Institute of Environmental Medicine, Karolinska Institutet, Box 210, Stockholm, 17177 Sweden; 3 Institute of Gerontology, School of Health and Welfare, Jönköping University, Jönköping, Sweden

**Keywords:** Centenarians, Longevity, Temporal trend, Population dynamics, Long-term care utilization, Polypharmacy, Morbidity

## Abstract

**Background:**

Centenarians have long been considered a select group who enjoy good health and manage to compress their years of morbidity towards the end of life. However, studies have shown that the health status of centenarians has changed as their numbers have increased. The aim of this study was to examine how the health of Swedish centenarians has changed in recent decades, focusing on healthcare utilization, medication use, morbidity, and mortality.

**Methods:**

Nationwide, register-based cohort study utilizing historical health records. All individuals born between 1890 and 1922 who turned 100 years old between 1990 and 2022 in Sweden were identified. We tracked and described their diseases prevalence, healthcare utilization, number of prescribed drugs prior to their 100 birthday, and one-year mortality risk at age 100.

**Results:**

The results reveal that over time, an increasing proportion of centenarians were diagnosed with chronic diseases. The prevalence of polypharmacy rose from approximately 50% in centenarians in 2006 to 80% in 2022. Concurrently, the proportion of centenarians residing in care homes declined, while the shares receiving formal home care or living without any formal care increased. One-year mortality remained rather stable over the period, with a slight decline for female centenarians.

**Conclusion:**

Overall, the data suggest that the health status of Swedish centenarians has worsened over time. Although some of these changes may reflect improved diagnostics and increased drug prescriptions, it is likely that morbidity has also risen as a result of greater survival from disease at older ages.

**Supplementary Information:**

The online version contains supplementary material available at 10.1186/s12877-025-06798-5.

## Introduction

Centenarians have long been considered a select group who enjoy good health and manage to compress their years of morbidity towards the end of life. However, as the population ages, the number of centenarians has also increased [1]. This might have lowered the level of health selection within this group. Improved survival with disease has driven much of the longevity improvements during the past decades, which may have resulted in a more heterogeneous centenarian population today compared to previous decades. Furthermore, evidence suggests that the mortality improvements observed in the general population have not extended to the very oldest individuals [2], and that centenarians of today exhibit a high level of polypharmacy and morbidity [3, 4]. This raises the question of how centenarians’ health profiles evolved over the past decades?

Few studies have examined the long-term trend in centenarians’ health status, and there is no consensus on whether their health status has improved or declined over recent decades. Some evidence suggests a compression of somatic diseases in centenarians compared to non-centenarians, though variation exists [1, 5, 6]. While the majority of centenarians are females, some studies have still explored gender difference. For example, later born cohort of centenarians have shown improvements in reported activities of daily living, especially in females [7]. Furthermore, survival advances beyond age 100 appear to be primarily driven by the longer survival of the healthiest individuals rather than those in poor health with female centenarians demonstrating greater robustness and independence over time [8]. In contrast, other research has reported that centenarians are becoming frailer over time, with females frailer than males [9]. This suggests an increasing reliance on assistance and care needs [10]. A recent Swedish study observed only 15% of centenarians lived at home without formal home care at the age of 100, while half lived in care homes [3]. Moreover, the rising number of centenarians has been associated with an increase in hospital admissions, particularly among females, as observed over a 16-year period in Spain [11]. In terms of prescription medication use, a Danish study found medication use increased both over time and with proximity to death among the oldest old. The prevalence of polypharmacy has also risen over time [12].

Despite extensive literature on various health profiles, research on long-term changes in centenarians’ health status at the population level remains scarce. With the growing number and proportion of centenarians, understanding how their health profiles have changed over time offers important insights for anticipating future healthcare needs, guiding the allocation of medical resources, and improving quality of life in aging populations. To address this gap, we conducted a nationwide population-based study to comprehensively examine changes in long-term care utilization, prescribed medication use, morbidity, and mortality among Swedish centenarians over a 30-year period.

## Methods

### Study population

All individuals born between 1890 and 1922 who turned 100 years old between 1990 and 2022 in Sweden were identified from the Total Population Register (TPR). Through the unique Swedish personal identification number, the Swedish Social Service Register (SSR), the Prescribed Drug Register (PDR), the National Patient Register (NPR), and the Cause of Death Register (CDR) were linked [13–16]. Due to different timing of register establishment, the availability of data varied. Data from the Prescribed Drug Register were available from 2006. Data from the SSR were available from 2012. The NPR reached national coverage in 1987. Details regarding the timeframes of each register are shown in Supplementary Figure S1.

### Measurement and statistical analysis

Information about long-term care utilization was extracted one year prior to the centenarians’ 100th birthday from the Social Service Register. In Sweden, long-term care is provided either in people’s homes (home care) or at care homes [17] and was defined accordingly. Centenarians were categorized as receiving “no formal care”, “receiving home care for less than 40 hours per month”, “receiving home care for 40 hours and more”, and “living in a care home.” Information on use of medical drugs was extracted from the Prescribed Drug Register one year leading up to their 100th birthday. The number of prescribed drugs was counted based on the 3rd level of the Anatomical Therapeutic Chemical (ATC) code. Centenarians were categorized into four groups based on number of dispensed drugs: 0 drugs, 1–4 drugs, 5–9 drugs, and 10 + drugs. Polypharmacy was defined as having five or more dispensed drugs of different kinds [18].

Chronic diseases were analyzed based on ten common conditions previously investigated among centenarians: cardiovascular disease (CVD), stroke, cataracts, chronic obstructive pulmonary disease (COPD), dementia, diabetes, non-skin cancer, renal disease, skin cancer, and thyroid disease. These diseases were identified from the National Patient Register using codes from the International Classification of Diseases (ICD) system versions 9–10. Detailed ICD codes are shown in the Supplementary Table S1. As the complete coverage of the National Patient Register began in 1987, and a five-year tracing back period was applied prior to each individual’s 100th birthday to identify diseases, the prevalence of chronic diseases was available from 1992. One-year mortality risk at age 100 was calculated as the number of centenarians who died during the year after turning 100 divided by the corresponding number of individuals. Date of death was obtained from the Cause of Death Register.

Sex-specific results were presented as proportion for each calendar year/birth cohort of centenarians and analyzed with descriptive statistics. All data were analyzed using R, version 4.4.2.

## Results

**Fig. 1:**
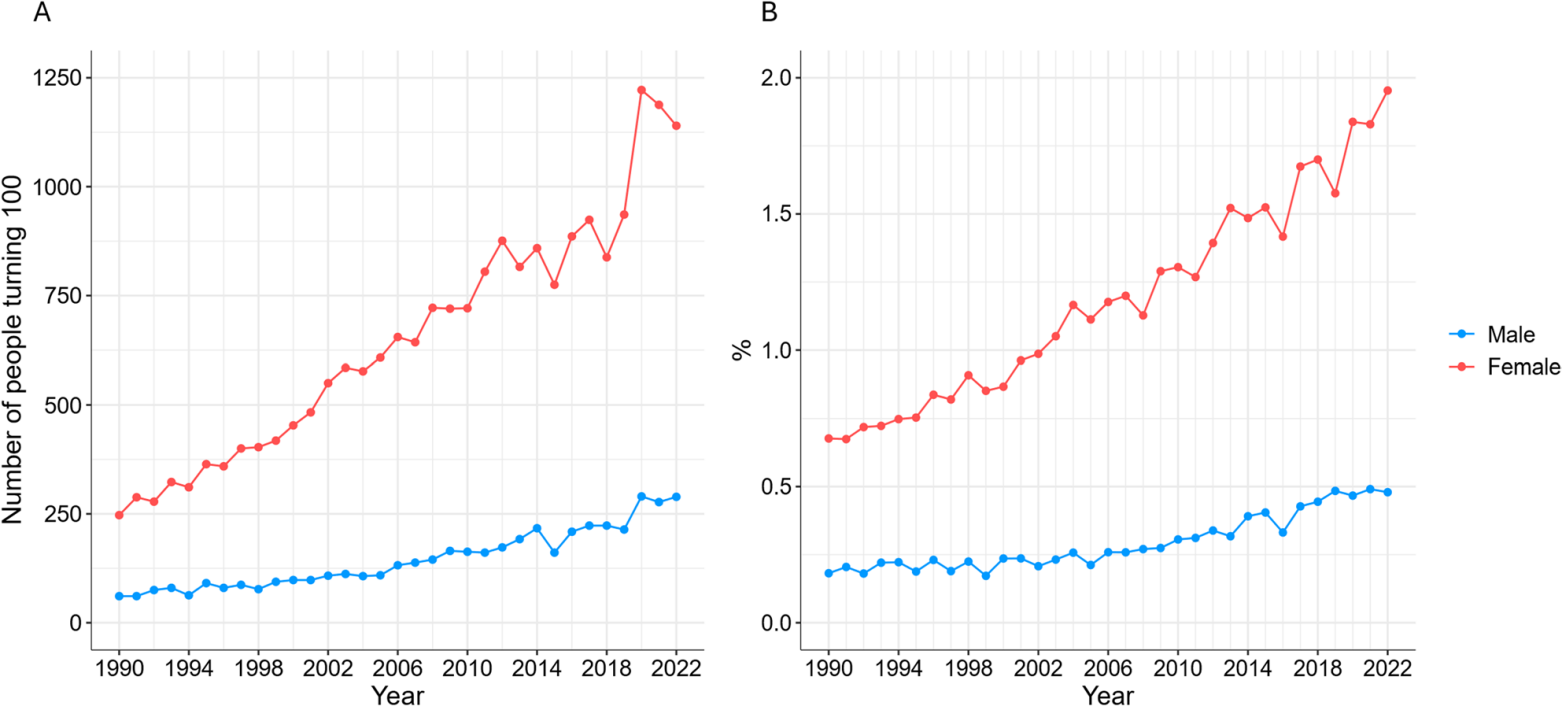
Number of Swedish centenarians (1990–2022) (**A**) and probability of surviving to age 100 by birth cohort (1890–1922) (**B**)

A total of 26,146 centenarians were included in the analysis, of whom 21,373 (81.7%) were female. The number of centenarians increased almost every year since 1990, with only minor declines observed in 2015, 2018, and post-2020. The number of centenarians in 1990 was 308, of whom 80.2% were female, and 1,429 in 2022, of whom 79.8% were female (Fig. 1). The probability of reaching 100 years old increased over time, with female having a consistently higher chance of surviving to age 100, and this discrepancy increased in the most recent decade. For female, the probability was 2.0% in 2022, while it was 0.5% for male (Fig. 1).

**Fig. 2:**
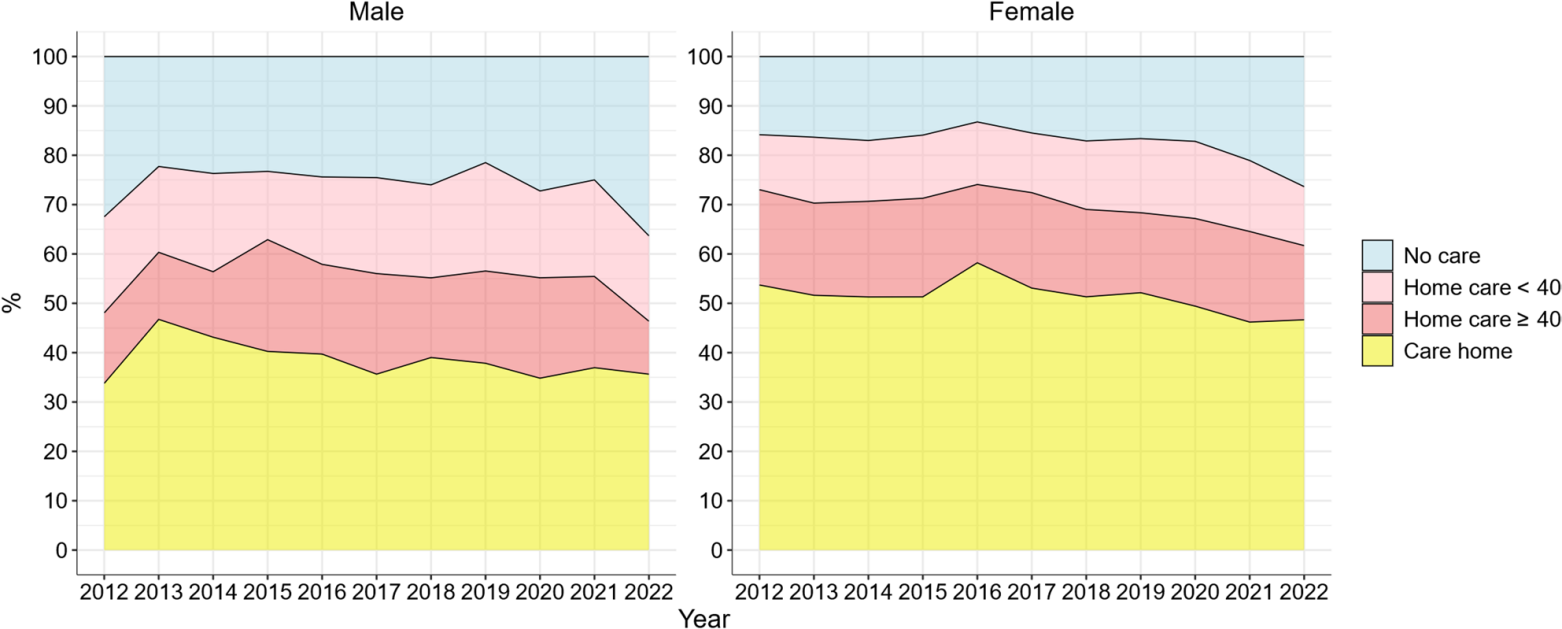
Care status of Swedish centenarians, 2012 to 2022. Note: The y axis represents the proportion of individuals with various amount of social care utilization

Figure 2 presents utilization of long-term social care among Swedish centenarians between 2012 and 2022. Overall, the share of centenarians without formal social care (blue in figure) increased over the last ten years, while the share of care home residence (yellow in figure) decreased. The proportion living at home with formal care (red in the figure) increased among male centenarians over time but remained relatively stable among females. A decline in this category was observed for both male and female centenarians in 2022. The proportion without formal home care increased from 18.5% to 28.4%. The relative increase was slightly larger in female centenarians (15.8% to 26.4%) than in male centenarians (32.5% to 36.3%), yet a higher share of male centenarians resided at home without any formal care. The proportion of centenarians residing in care homes declined from 50.5% to 44.4%, and the decline was slightly larger in female centenarians (53.7% to 46.7%) than in male centenarians (which fluctuated but generally remained stable at around 35% from 2012 to 2022). Notably, female centenarians consistently had higher proportion of residing in care homes and less likely to live without formal care, compared to male centenarians.

**Fig. 3:**
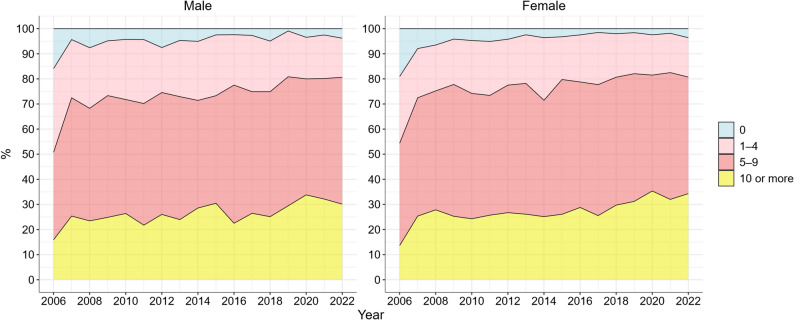
Dispensed drugs in Swedish centenarians, 2006 to 2022. Note: The y-axis represents the proportion of centenarians with different number of dispensed drugs. The register was introduced in July 2005; 2006 was the first full calendar year. The increase between 2006 and 2007 may partly reflect register introduction and stabilization

Figure 3 presents the proportion of different numbers of drug prescriptions for Swedish centenarians between 2006 and 2022. Overall, both the number of prescribed drugs and the proportion of polypharmacy increased with time. The average number of prescribed drugs in female centenarians increased from 5.1 to 8.0, and from 5.2 to 7.8 in male centenarians (not shown in figure). The proportion of polypharmacy increased from 54.4% to 80.7% in female centenarians, and from 50.7% to 80.6% in male centenarians.

**Fig. 4:**
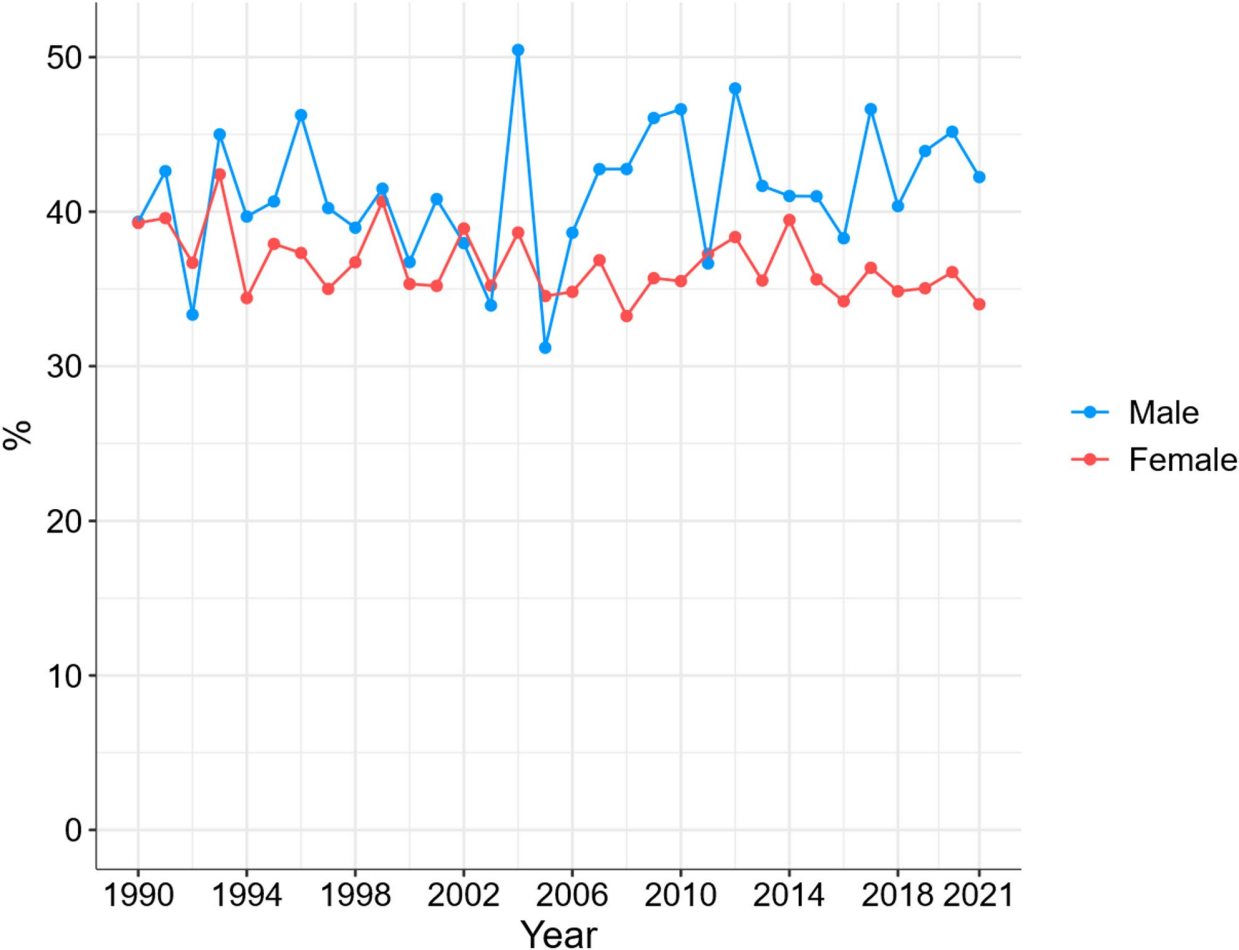
One-year mortality risk of Swedish centenarians, 1990–2021. Note: Every centenarian was given the chance of surviving one year after reaching age 100, meaning that the last year 2021 represents survival for individuals turning 100 years in 2021

Figure 4 presents the one-year mortality risk for Swedish female and male centenarians from 1990 to 2021. It was fairly stable during the past 30 years at approximately 40% for male centenarians and around 35% for female centenarians, although the mortality went down slightly over time for female centenarians.

**Fig. 5:**
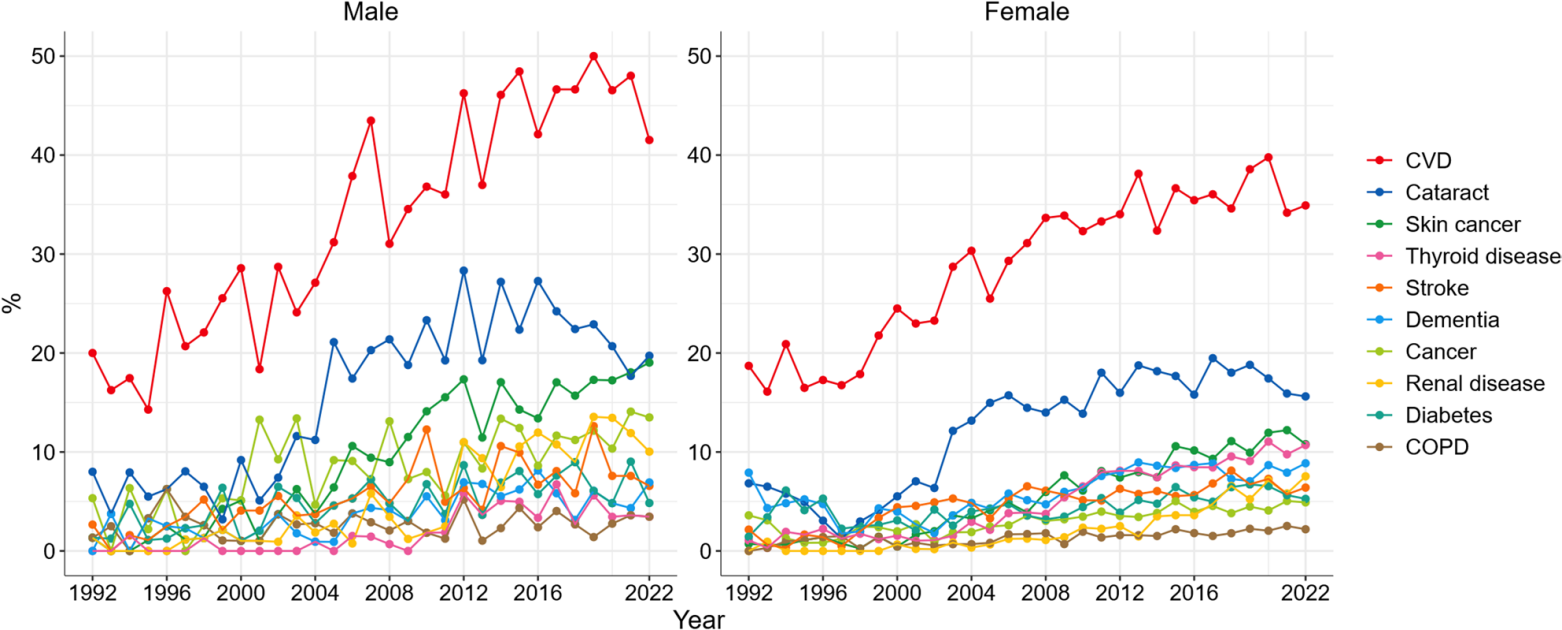
Prevalence of diseases among Swedish centenarians, 1992–2022

Figure 5 shows the prevalence of chronic diseases among Swedish female and male centenarians. Overall, the prevalence of all conditions increased over time, with greater fluctuations observed among male centenarians. CVD and cataract remained the most prevalent conditions throughout the study period, showing relatively stable increase. In contrast, more pronounced increases were observed for thyroid disease among females and renal disease among males. Specifically, the prevalence of thyroid disease among female centenarians rose from 0.7% in 1993 (0% in 1992) to 10.7% in 2022, a more than 15-fold increase. Among male centenarians, the prevalence of renal disease increased from 1.6% in 1993 (0% in 1992) to 10.0% in 2022, representing a more than 6-fold increase. Except diabetes, thyroid diseases and dementia, the prevalence of other chronic conditions appeared higher in male than in female centenarians.

## Discussion

This study explored how levels of long-term care, number of dispensed drugs, prevalence of chronic diseases, and one-year mortality risk have changed among Swedish centenarians over the past three decades. We hypothesized that the growing number of centenarians may have diluted the health selection that characterized earlier cohorts, making today’s centenarians more similar to the general older population. Our findings provided both support for and rejection of this hypothesis, depending on the health measure considered. The centenarian population today indeed have higher levels of polypharmacy and diagnosed diseases compared with previous decades. Among female centenarians, the one-year mortality risk has slightly declined to approximately 35%, whereas among male centenarians, it has remained stable at around 40% with no clear downward trend. Regarding long-term care utilization, the proportion of centenarians living in care homes has decreased over time, while the proportions receiving home care have increased. This shift likely reflects the impact of aging-in-place policies rather than improvements in health [19]. Nevertheless, the majority of centenarians still receive long-term care, which aligns with previous research on long-term care distribution among Swedish centenarians [3]. However, our study is the first to describe the temporal trends in long-term care utilization among centenarians in Sweden over a ten-year period. The higher proportion of male centenarians without formal long term care compared to females may partly be explained by the fact that males are more likely to cohabit and therefore more likely to receive informal care. Notably, the share without any formal care remained fairly stable over the period but increased since 2020 for both female and male centenarians. This shift may be partly attributed to the COVID-19 pandemic and a drop in elder care admissions during these years.

Notably, there is no apparent spike in mortality risk observed during the pandemic years. We believe this could be explained by several factors. First, mortality is high in centenarians with a remaining life expectancy at age 100 of around two years [20]. Thus, the relative impact of COVID-19 on centenarian mortality may be less visible compared to younger age groups. Even though some centenarians were affected, COVID-19 could have replaced other causes of deaths without affecting overall mortality much. An alternative explanation is that centenarians may be more resilient to infectious diseases [21]. Centenarians have shown capable of generating high levels of neutralizing antibodies and experiencing relatively mild symptoms, perhaps due to early-life infectious pandemics (such as the 1918 influenza), better regulation of immunoinflammatory responses, or distinct gut microbiome profiles that may support immune function [21, 22].

The observed increase in polypharmacy can be be attributed to several factors, one of them is the improved survival following acute and chronic diseases, which may have led to a growing prevalence of chronic conditions over time and thus more medications [4, 23]. Although the introduction and stabilization of the prescription register may have contributed to the apparent increase in drug prescription between 2006 and 2007, this does not undermine the overall long-term increasing trend. The high prevalence of polypharmacy among centenarians is also consistent with findings from previous studies [24, 25]. While the increase in drug use could be a result of change in attitude of providers treating centenarians as other older adults in terms of prescription rates, it is important to note that they have a short remaining life expectancy and that severe polypharmacy may have negative effects, not only among centenarians but among older people in general [26, 27]. Drug-drug interactions, adverse drug reactions, and drug-disease interactions may compromise quality of life [26, 28]. Therefore, distinguishing appropriate and inappropriate polypharmacy and careful considering prescribing practices in this population group is important. Encouragingly, randomized clinical trials in recent years have begun to evaluate the effectiveness of patient-centred deprescribing interventions in older adults [29].

The increasing prevalence of chronic diseases may partially be attributed to ‘diagnostic drift,’ as more people are diagnosed due to advances in medical equipment and diagnostic precision over time, such as the great increase in thyroid disease. However, it should be noted that for most diseases, we observed an increase also in the most recent years, where diagnostics have been rather unchanged. Moreover, improved treatments have led to more individuals surviving longer with diseases, allowing those who may not have survived in the past to become centenarians today.

Since 1990, many high-income countries have documented declines in both the incidence and prevalence of CVD among older adults [30]. Nevertheless, the overall burden of CVD remains considerable in aging populations [30]. We also observed a high CVD burden, and CVD showed the greatest increase in prevalence over time. Yet centenarians experience lower incidence and risk of severe CVD events—such as stroke and myocardial infarction—than their shorter-lived peers, suggesting a degree of survival selection [6, 31].

Another Swedish study found a higher prevalence than we did [32]. The substantially higher prevalence observed in that study compared with our findings may partly reflect differences in case definitions, methods for calculating prevalence, and characteristics of the study populations. For example, in our study, CVD and stroke were analyzed as separate conditions across all Swedish centenarians. Comparable studies in other populations have similarly reported high CVD prevalence among centenarians [1, 4].

Apart from CVD, cataracts are another common condition with a significant prevalence increase among centenarians in recent decades. Age-related cataracts are the leading cause of reversible vision loss worldwide and account for an estimated 46% of all blindness cases, substantially impairing quality of life in older adults [33]. Although the prevalence of cataracts has increased among centenarians over decades, centenarians experience lower incidence and prevalence of cataracts compared to non-centenarians [25].

Over the past 30 years, female centenarians have generally shown a lower prevalence of most chronic diseases compared to their male counterparts, with the exceptions of dementia, diabetes and thyroid disease. These sex differences may reflect variations in lifestyle factors as well as underlying biological mechanisms, including hormonal influences, genetic factors and immune responses [34, 35]. There is no consistency regarding the morbidity burden among female or male centenarians [3, 25, 36]. Some studies have reported that female centenarians exhibit higher morbidity than males, a pattern often attributed to selective survival, whereby males with comparable health conditions are less likely to survive to age 100, leaving a relatively healthier male population at this age [1, 37, 38]. However, with the improved survival from diseases over time, more people survive longer and are more likely to become centenarians, which means centenarians may be increasingly heterogeneous. This potentially reduces the effect of selective survivor bias in male centenarians.

### Strengths and limitations

The main strength of the current study is its population-wide coverage, enabling inclusion of all Swedish centenarians from 1990 to 2022 with health information from everyone without selection. The main limitation of the study is that we are not able to account for the influence of contextual changes over time without contemporaneous comparison groups. Although strokes in 1990 and 2024 are both categorized as a stroke, improvements in diagnostic precision over time makes it likely that milder and minor strokes are detected to a higher degree today compared to the past. Thus, the increasing trend may therefore partly reflect contextual developments rather than true shifts in population health. Yet, diagnosis shifts only partly explain the actual reasons behind the observed increase. Increased survival of individuals with diseases who would have previously died of chronic morbidity plays a role in this upward trend of morbidity [39]. Furthermore, the validity and quality of Swedish register data are high, and contextual changes may not undermine the overall trends observed [15, 40]. Another limitation is that the included registers have varying follow-up periods, which restricted the analysis of trends in drug prescriptions and long-term social care utilization to a shorter period. Moreover, we did not examine which types of medical drugs accounted for the prescriptions. However, a previous study of Swedish centenarians has shown that commonly prescribed medications are related to pain management (analgesics) and heart failure (diuretics) [3]. Additionally, primary care data are not available for the current study. Access to such data could have provided more information such as drug prescriptions on conditions that were previously managed in primary care but are now increasingly treated in specialized care, such as dementia and diabetes. Finally, somatic diseases and drug use capture only one dimension of health. Although long-term care utilization may partly reflect functional capacity, additional data on physical function, cognitive status, and social engagement are needed to more comprehensively characterize shifting health trends in the centenarian population.

## Conclusions

A comparison of several indicators of morbidity and care needs among Swedish centenarians over time reveals both improvements and potential deteriorations in their health. The slightly higher survival of female centenarians today compared with earlier cohorts, along with the modest increase in the proportion living at home without formal long-term care, suggests improvements in centenarians’ overall health. In contrast, centenarians show higher levels of polypharmacy, and an increasing prevalence of diagnosed diseases today compared to the past suggests deteriorations in health status. While part of the rise in disease prevalence can be attributed to improved diagnostics, it may also reflect increased survival with chronic conditions. Future research should focus on identifying factors that delay the onset of multiple diseases in old age, and, considering the high levels of polypharmacy, explore whether deprescribing certain medications could improve centenarians’ quality of life and care.

## Supplementary Information

Supplementary Material 1.

## Data Availability

The individual level data underlying this study cannot be shared publicly because of the General Data Protection Regulation in Sweden. Access to the data and statistical code can be permitted to external researchers after ethical vetting and establishment of a collaboration agreement. Contact the corresponding author for questions about data sharing.

## Abbreviations

ATC: Anatomical Therapeutic Chemical code
CDR: Cause of Death Register
COPD: Chronic obstructive pulmonary disease
CVD: Cardiovascular disease
ICD: International Classification of Diseases
NPR: National Patient Register
PDR: Prescribed Drug Register
SSR: Social Service Register
TPR: Total Population Register
