# Supplementary material for: Swedish centenarian health – a nationwide, observational study on care utilization, drug use, morbidity, and mortality among Swedish centenarians in 1990 to 2022
Source: BMC Geriatr. 2025 Nov 25;25:1011. doi: 10.1186/s12877-025-06798-5 (PMC12687530; doi:10.1186/s12877-025-06798-5)
Supplement: Supplementary file 1 — Supplementary Material 1. [file 12877_2025_6798_MOESM1_ESM.docx]

Table S1 Swedish ICD9 and ICD10 codes used to identify diseases

| Disease | ICD9 | ICD10 |
| --- | --- | --- |
| Cardiovascular disease | 410; 411; 413; 4273; 4280; 430; 431; 4320; 4321; 4329; 4330; 4331; 4332; 4333; 4338; 4339; 3448; 4340; 4341; 4349; 436; 4376; 4370 | I20; I21; I22; I48; I50; I60-I64 |
| Diabetes | 250 | E10-E14 |
| Stroke | 430; 431; 4321; 4320; 4329; 4330; 4331; 4332; 4333; 4338; 4339; 3448; 4340; 4341; 4349; 436; 4376; 4370 | I60-I64 |
| Cancer (not skin) | 140; 141; 142; 143144; 145; 146; 147; 148; 149; 150; 151; 152; 153; 154; 155; 156; 157; 158; 159; 160; 161; 162; 163; 164; 165; 166; 167; 168; 169; 170; 171; 174; 175; 176; 177; 178; 179; 180; 181; 182; 183; 184; 185; 186; 187; 188; 189; 190; 191; 192; 193; 194; 195; 196; 197; 198; 199; 200; 201; 202; 203; 204; 205; 206; 207; 208 | C except for C43 and C44 |
| Skin cancer | 172; 173 | C43; C44 |
| Thyroid disease | 2400; 2409; 2410; 2411; 2419; 2420; 2421; 2422; 2423; 2424; 2428; 2429; 243; 2443; 2448; 2449; 2450; 2451; 2452; 2454; 2453; 2458; 2459; 2460; 2461; 2462; 463; 2468; 2469 | E03; E04; E05; E06; E07 |
| Renal disease | 403A; 403B; 403X;582; 583; 585; 586; 588A; V42A;V45B;V56 | N032-N037; N052-N057; N11; N18; N19; N250; I120; I131; Q611-Q614; Z49; Z940; Z992 |
| Dementia | 290; 294B; 331A; 331B; 331C; 331X | F00-F03; F051; G30; G311; G319 |
| COPD | 491; 492; 496 | J43; J44 |
| Cataracts | 3660; 3661; 3662; 3663; 3664; 3665; 3668; 3669; 3793 | H25; H26; H28 |


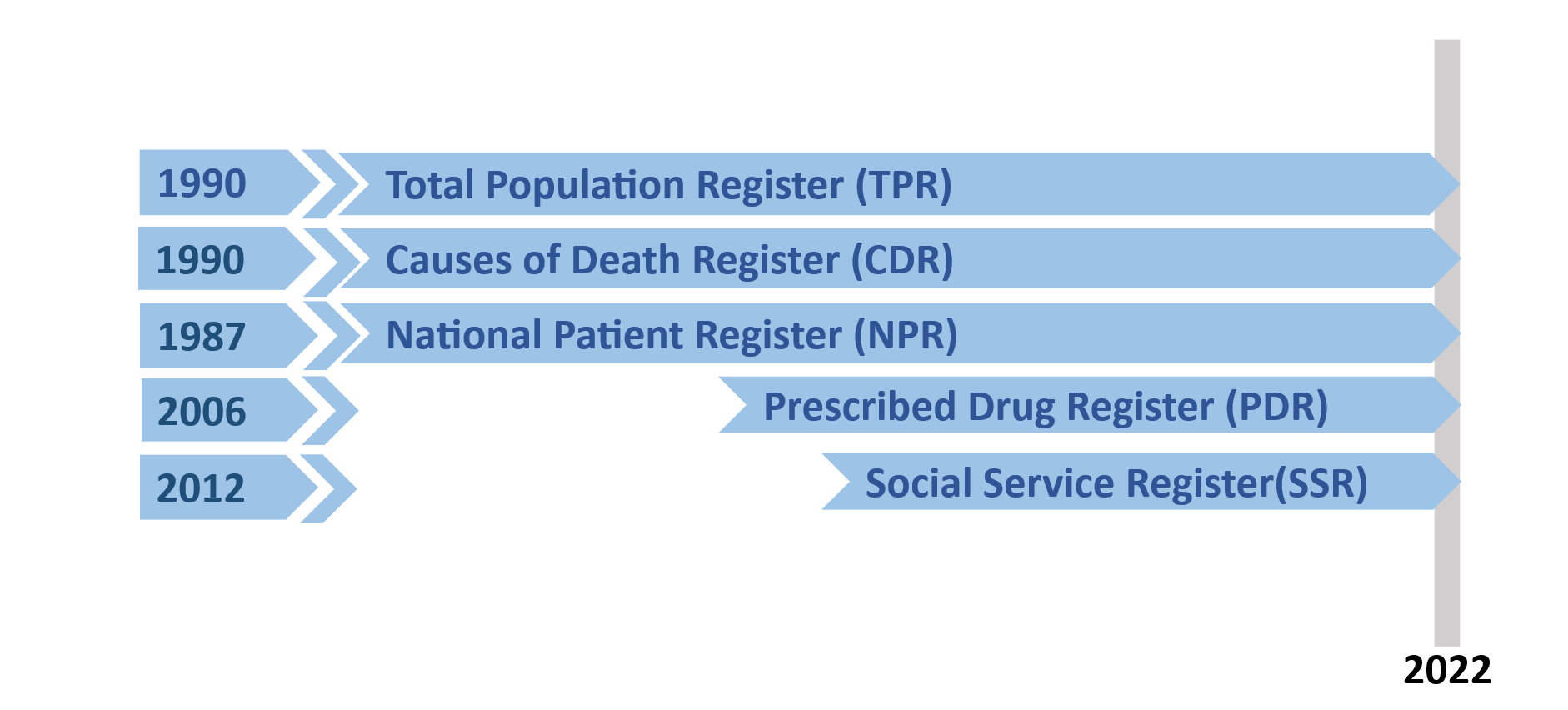


Figure S1 Timeframes of Swedish national registers
